# Getting DBT online down under: The experience of Australian and New Zealand Dialectical Behaviour Therapy programmes during the Covid-19 pandemic

**DOI:** 10.1371/journal.pone.0275636

**Published:** 2022-10-06

**Authors:** Emily B. Cooney, Carla J. Walton, Sharleen Gonzalez

**Affiliations:** 1 Yale University, New Haven, Connecticut, United States of America; 2 University of Otago, Wellington, New Zealand; 3 Hunter New England Mental Health Service, Newcastle, New South Wales, Australia; The Royal Melbourne Hospital, AUSTRALIA

## Abstract

Dialectical Behaviour Therapy (DBT) is an intensive and multi-modal intervention developed for individuals with multiple comorbidities and high-risk behaviours. During pandemic-related lockdowns, many DBT services transitioned to delivering treatment via telehealth, but some did not. The current study sought to explore the experience of DBT teams in Australia and New Zealand who did and did not transition to telehealth during the early stages of the COVID19 pandemic, as the majority of research on DBT via telehealth has originated from North America, and focussed on therapists who did make this transition. DBT team leaders in Australia and New Zealand completed a survey with open-ended questions about the barriers they encountered to delivering DBT via telehealth, and for those teams that implemented telehealth, the solutions to those barriers. Respondents were also asked about specific barriers encountered by Indigenous and Pacific people service users. Of the 73 team leaders who took part, 56 reported providing either individual therapy, skills training or both modalities via video-call during lockdown. Themes emerging from perceived barriers affecting just DBT providers included the assessment & management of emotions and high-risk behaviours, threats to privacy and information security posed by telehealth, logistical issues related to remote sessions, and the remote management of therapy-interfering behaviour. Themes emerging from perceived barriers affecting both providers and service users included disruptions to therapeutic alliance, lack of willingness, lack of technical knowledge, lack of private spaces to do DBT via telehealth, and lack of resources. The solutions most frequently cited were the provision of education and training for therapists and service users in the use of telehealth, and the provision of resources to access telehealth. These findings are relevant to clinical delivery of DBT, as well as planning and funding for DBT telehealth services.

## Introduction

Dialectical behaviour therapy (DBT) is an intensive psychotherapy that was originally developed for individuals with borderline personality disorder (BPD), characterised by chronic emotion dysregulation, self-injury and suicidal behaviour [1]. Initially developed for outpatient settings, it is comprised of a consultation team for therapists, individual sessions, skills training typically provided in group sessions, and between-session coaching [2]. Due to the intensive nature of DBT treatment, it has typically been provided as a face-to-face only service by most providers.

However, the COVID-19 pandemic set in motion an accelerated shift to provide many mental health services via telehealth for significant periods of time across a range of populations and settings [3, 4]. Telehealth has been defined as “the delivery of healthcare services, where distance is a critical factor, by all healthcare professionals using information and communication technologies for the exchange of valid information for diagnosis, treatment and prevention of disease and injuries” [4]. Increasing patient access to evidence-based and comprehensive mental health treatments through telehealth is important both during the pandemic and beyond. Global prevalence estimates indicate that over 93 million adults meet criteria for BPD worldwide [5]. Research on the ratio of specialist treatment providers to treatment-seeking individuals with BPD indicates that demand far exceeds supply [6]. Telehealth is an essential part of the solution for this urgent public health problem [7]. However, to implement this, healthcare systems need to know more about the barriers to providing DBT via telehealth, and generate solutions for these.

Through necessity born of the COVID19 pandemic, a worldwide proliferation of virtual DBT programs has outpaced the data [8]. Early papers on the transition to DBT telehealth involved practitioner opinions, or used qualitative methodology focussing on the experience of DBT service providers to identify a number of challenges and solutions [9–13]. Zalewski and colleagues surveyed 200 DBT providers based mostly in the US who had pivoted to delivering DBT via telehealth. The survey did not differentiate between respondents based in fee-for-service versus government-funded programs. Lessons learned by clinicians included the benefits of using DBT skills and strategies on oneself to minimize the risk of burnout, the importance of taking a dialectical and principle-based approach to adherence to the model, and the need for proactive targeting of therapy-interfering behaviours during telehealth [9] which were echoed by smaller survey-based and anecdotal accounts of delivering DBT via telehealth [10, 11, 13].

While many DBT providers navigated telehealth service delivery [13–17], some did not. An early Australian survey of government-funded mental health services indicated that the majority of respondents in that particular region had not transitioned to online DBT during the COVID19 pandemic [12], reporting low confidence in delivering DBT online, and anticipating that clients would have insufficient resources to do so.

A limitation of the studies above is that they focused on the experiences of DBT programs which did transition to DBT via telehealth, or those which did not, rather than canvassing all DBT programs across a geographical region to learn more about the factors that influence the decision whether or not to provide services via telehealth. One possible factor to consider is service funding. Services with more independence could respond more flexibly to the demands of the pandemic, whereas programs within government-funded mental health services may have had less freedom or perceived autonomy to do the same. In addition, clients of government-funded services may have had less resources needed at home to access telehealth services.

Having a better understanding of the factors that blocked or facilitated the launch of DBT telehealth programs is critical for three reasons. Firstly, telehealth is highly relevant to the goal of improving access to mental health services across all communities [7]. Secondly, any solution to access challenges holds particular relevance for vulnerable communities experiencing mental health inequities, particularly those for whom distance or transportation problems may affect access. Gathering more nuanced information on the impact of telehealth services on health disparities experienced by marginalised communities, such as Indigenous peoples and people living in rural and remote areas, is critical to expanding access for these communities. Thirdly, understanding the barriers to DBT delivered via telehealth that have been experienced as insurmountable by some services provides the detailed assessment information that is necessary for the development of targeted solutions that will actually resolve the barriers within those contexts.

This consideration may be particularly relevant for Indigenous communities. In both Australia and New Zealand, Indigenous peoples experience significant health and economic disparities [16–19]. Evidence suggests that in both Australia and New Zealand, a greater proportion of Indigenous people live in rural or remote areas [19, 20], and would therefore have less access to DBT services. Information about digital access for Indigenous and Pacific individuals in Australia and New Zealand is limited [21]. Research into barriers to access can be used to advocate for systemic change at policy levels to facilitate DBT delivered via telehealth in a way that might reduce rather than maintain systemic marginalisation and racism in healthcare.

The current study sought to build and expand on previous work by identifying the percentage of surveyed teams in Australia and New Zealand who transitioned to telehealth and exploring the perceptions of DBT team leaders in Australia and New Zealand on barriers and solutions to the provision of DBT delivered via telehealth in their service. Both countries have well-established DBT programs in multiple regions, and DBT is represented in both the public and private health sectors. By inviting DBT team leaders of programs that did pivot to telehealth, and those of programs that did not, the hope was to gain a more nuanced understanding of the factors hindering DBT delivered via telehealth implementation, and solutions to these barriers. Additionally, the study sought team leaders’ perceptions of barriers to DBT telehealth experienced by Indigenous and Pacific peoples in their services.

## Materials and methods

The data reported here were collected as part of a larger study. The study was conducted in both Australia and New Zealand, with regional ethics approval provided by the Hunter New England Human Research Ethics Committee in Australia (Reference 2020/ETH02299) and University of Otago Human Research Ethics Committees in New Zealand (Reference HD20/109).

### Recruitment

A snowballing technique was used to send out study invitation emails to DBT team leaders of services in Australia and New Zealand delivering DBT that provided individual sessions, group skills training and/or team consultation. Emails were sent to DBT programs known to the researchers and those identified through online DBT registries via Google. The invitation email included a link to a brief RedCap survey preceded by a digital consent process and encouragement to share the email with other DBT providers.

The survey included questions about the components of DBT offered by services, whether and how these were delivered during the first lockdown of the year 2020 occurring in their region, and the barriers and solutions experienced by the service in delivering DBT via telehealth. ‘Lockdown’ was defined as “the time when the government advised significant restrictions and minimal movement unless necessary to slow the spread of COVID-19 in your area.” The first email was sent out in December 2020 and the survey was available to complete until May 2021.

### Design

The study used a cross-sectional mixed-method design.

### Participants

Respondents were included if they were the team leader of a mental health centre, service or clinic that provided DBT individual sessions, and/or group skills training and team consultation for adults or adolescents in Australia or New Zealand prior to lockdown.

### Materials

The survey questions related to the focus of this paper are summarised in this section. See the S1 File for a full copy of the survey.

The first part of the survey involved questions on demographics and DBT service delivery. The second part explored changes made to treatment delivery during lockdown. This included whether DBT modalities were delivered via telehealth, barriers to providing telehealth, solutions to these barriers, whether any clients who were unable to participate in DBT via telehealth identified as Indigenous or Pacific People, and perceived barriers for those service users.

For each of the pre-lockdown modalities endorsed by respondents, they were asked whether they continued to provide this during lockdown. Respondents reporting yes were asked “What barriers did your centre encounter to providing [endorsed modality] via telehealth during COVID-19’s lockdown period?” and then “What solutions did you arrive at to overcome these barriers?” Respondents reporting no were asked “What were the barriers to your centre providing [endorsed modality] via telehealth during COVID-19’s lockdown period?”

Later in the survey, respondents reporting that they had offered telehealth, were also asked whether there were any individuals who were not able to participate in telehealth. Respondents reporting ‘yes’ to this question were asked “Did any of these clients identify as Aboriginal or Torres Strait Islander or Māori or Pasifika?”, followed by the open-ended question “What were the barriers? Please specify if any of the barriers were more likely to be experienced by Aboriginal or Torres Strait Islander or Māori or Pasifika clients.”

### Analysis

Analysis involved two processes. First, responses to open-ended questions on barriers and solutions to individual therapy and group skills training were coded into themes separately by two of the three authors (EBC & SG, with the exception of barriers to individual therapy which was coded by EBC & CJW). This involved an iterative process and inductive approach drawing on grounded theory analytic strategies of comparative analysis [22]. Initially, all responses were reviewed by the first author (EBC) to identify potential themes from the data. This was followed by a granular coding process, identifying the micro-themes within statements and noting the frequency with which these occurred. From this bottom-up approach, broader themes emerged that encompassed the specific detailed codes identified earlier. Independently, the two other authors also examined responses within the categories as described above and identified the themes they saw arising from the data, taking a top-down approach. Further collaborative discussion focussed on developing a consensus regarding the overarching themes of the data, and reaching agreement on the illustrative quotes for the main themes arising from responses.

The second part involved two of the authors (EBC & SG) independently recoding responses, and calculating frequencies to identify the four most-cited barrier and solution themes. Where divergence occurred, the second author (CJW) independently reviewed the quotes to facilitate a consensus on interpretations of themes.

Responses to the open-ended question regarding specific barriers for clients who were unable to participate in telehealth were also coded with specific attention to the team leaders’ perceptions of barriers experienced by Indigenous and Pacific peoples.

## Results

Thirteen of the 17 NZ teams (76%) and 60 of the 141 (43%) Australian teams who were approached completed the survey. Of those who completed the demographic questions, the majority (61 i.e. 86%) led outpatient DBT programs. The remainder led day hospital programs. The majority of teams were embedded within government-funded mental health services (58%, n = 42). Others worked within for-profit private practice clinics (19%, n = 14), private hospitals (11%, n = 8) and Non-Government/not-for-profit clinics (12%, n = 9). Most teams (58%) worked solely with adults, some teams worked with adolescents (15%), and others worked with both age groups (27%). Three teams indicated that they began providing DBT programs during lockdown. Estimates of the total number of clients attending DBT in any mode of treatment for programs before COVID-19 yielded an average of 21.8 (range 0–100, SD = 20).

Table 1 shows the numbers of teams providing each DBT modality before and during lockdown; group skills training was the most prevalent modality, both before and during lockdown, including via video-conferencing during lockdown. The majority of respondents (56 i.e. 77%) indicated that they had used video-conferencing to assist with providing at least one DBT modality (individual sessions or DBT skills training) via telehealth. Of note, nine respondents (12%) reported that their teams did not offer individual therapy or skills training in person or via telehealth during this time. Forty respondents provided estimates of the total number of clients attending any mode of DBT treatment for their program at the time of survey completion. This yielded an average of 13 (range 0–70, SD = 16). Sixty respondents provided an estimate of the number of groups typically run at their service at the time of survey completion, which yielded an average of 2 groups (range 0–13, SD = 2).

**Table 1:** Modalities provided before and during lockdown by respondents’ DBT teams.

Modality	Before Lockdown	During Lockdown	During lockdown–video-conferencing
Individual Therapy	53 (73%)	47 (64%)	42 (58%)
Skills Training	73 (100%)	54 (74%)	45 (62%)
Consultation Team	57 (78%)	53 (73%)	41 (56%)

Approximately half (37) of the team leaders indicated that some clients were unable to access DBT via telehealth, and of these, 9 team leaders indicated that within this group, there were individuals who identified as either Indigenous or Pacific people.

Tables 2 and 3 show the main themes emerging from responses to open-ended questions on barriers and solutions regarding the remote delivery of individual DBT and skills training. At a broad level, the themes arising from team leaders’ responses tended to fall into two categories: (1) perceived barriers relating specifically to therapists (which encompassed individual as well as organisational/systemic obstacles) as shown in Table 2, and (2) perceived barriers relevant for both clients and therapists as shown in Table 3. Pragmatically, responses are framed in terms of the themes arising from the barriers which then drove solutions.

**Table 2:** Perceived barriers for therapists in delivering DBT via telehealth, and associated solutions.

Perceived Barrier	Solutions
Assessment & management of emotions and high-risk behaviours, particularly behaviours generating concerns about safety	Development of telehealth safety protocols Clinicians liaise and communicate more regarding suicide and self-harm risks with service user and others Service to check that they have access to all clients’ contact details Some clinicians may be mindful of avoiding distressing topics if the client seems already distressed
Privacy and information security concerns	Service to research security of various video call platforms Managers and clinicians to advocate to use telehealth despite privacy concerns to improve access for clients Development and distribution of information sheets and agreement forms on limits and risks of telehealth for clients
Logistical challenges related to non-physical meetings	Clinicians to mail out copies of handouts and worksheets ahead of time Clients can email completed materials ahead of therapy, or clients can hold completed materials up to camera Clinicians to tailor activities to online format in choice of exercises and use of media Clinicians to tell clients what to bring to session Clinicians need to set more time aside for pre-session preparation and orientation activities for clients
Managing therapy-interfering behaviour is harder via remote platforms	Service to create guidelines describing expectations of therapy via telehealth Clinicians to use more engagement strategies and send out more email/text reminders to maintain engagement Clinicians to target problem behaviour in individual and skills sessions Service may have an additional facilitator in telehealth group skills training sessions to manage clients’ therapy-interfering behaviours Clinicians can use breakout rooms to coach individuals Clinicians can reach out more frequently between sessions

**Table 3:** Perceived barriers for both therapists and clients in delivering DBT via telehealth, and associated solutions.

Disruptions to therapeutic alliance	Clinicians to acknowledge and discuss the problem with clients Clinicians to validate and increase use of phone, email and text to improve therapeutic alliance Clinicians can encourage more chat and socialising in groups during group breaks Clinicians to be more animated and exude more warmth during individual therapy and group sessions Clinicians to encourage group members to reach out to each other during breaks Clinicians can request consent for clients to share their completed homework sheets with the group
Lack of willingness	DBT teams can problem-solve in consult meetings, and explore reasons for unwillingness amongst staff and clients Clinicians can role-model giving telehealth a try and invite fellow team members and clients to do the same Teams can highlight freedom to choose in the absence of desirable alternatives to DBT telehealth, to both clients and fellow DBT team members Service can validate staff and clients struggles Service can provide information about others’ positive experience of telehealth Service can resource administrative staff to encourage clients to schedule a first telehealth session
Lack of technical knowledge	Service to increase tech support to both clients and therapists Service or managers to educate and train staff in telehealth and the video call program of choice Identify and support telehealth clinician ‘champions’ who trial, troubleshoot and model solutions, and then feedback to the rest of the team Clinicians to orient and coach clients Development of tip sheets for clinicians and clients
Lack of private spaces to do DBT via telehealth	Encourage use of headphones for clinicians and clients Increase access of individual devices and spaces for clinicians where possible, improve resources for clinicians from a service-level Actively problem-solve and use flexibility re location of therapy sessions for privacy (e.g. cars) if no other private spaces are available Provide the option of private rooms in GP or other community service space for clients
Lack of resources	Managers and clinicians to advocate for provision of software, hardware and connectivity for clinicians and clients Use ethernet rather than wifi to improve internet connection

Some themes were specific to one particular modality, and others overlapped across both individual therapy and group skills training. Overarching themes that arose regarding perceived barriers for both clients and therapists included access to the practical resources needed to provide DBT via telehealth (‘resources’) and concerns related to the impact of telehealth on doing the actual therapy, such as finding telehealth aversive, the loss of reinforcement associated with in-person connection, assessment and management of suicide risk and therapy-interfering behaviour (‘process’). Two participants reported their teams didn’t encounter any significant barriers to providing DBT over telehealth other than initial minor technical issues.

The most frequently cited barriers were: practical technological issues and resource deficits, engagement and rapport issues between clinicians and clients, and logistical difficulties providing telehealth services associated with the mechanics of doing therapy remotely. The most frequently cited solutions were: educating and training clinicians and clients in telehealth delivery, engaging willingness for clinicians and clients, remote logistics solutions and addressing resources issues.

Seven team leaders indicated their teams did *not* provide individual therapy via telehealth during lockdown. Reasons included concerns about the assessment and management of risk via telehealth, threats to privacy, and clients not having devices to connect. A total of 23 team leaders indicated their teams did *not* provide group skills training via telehealth during lockdown. Themes emerging from these included lack of resources (access to software and hardware), lack of technical knowledge (and consequent lack of confidence in using the technology), and lack of willingness for clinicians and clients, partly fuelled by the absence of research on DBT via telehealth. In addition, four of the leaders of teams who did not provide DBT via telehealth reported that their managers prohibited the delivery of therapy (most frequently group sessions) via telehealth.

### Perceived barriers for therapists

#### Assessment and management of emotions and high-risk behaviour

Respondents who provided DBT via telehealth and those who did not mentioned the remote assessment and management of emotion dysregulation and risk concerns as a major barrier in both individual therapy and group skills training via telehealth:

*“Losing more subtle interpersonal interactions*.*”*^1–99^

*“there were initial concerns around the management of risk (unable to complete risk assessments in person or at the clinic, if people dropped offline if was more difficult to manage initially then if someone walks out in a group—you can follow up if they don’t return)”* ^1–41^

*“Assessing group engagement and monitoring participants affect / coping in sessions is difficult when you can’t see everyone on the same screen*.*”*^2–31^

Solutions included the development of protocols to manage safety concerns during remote therapy, increased communication with the individual and others regarding risk, ensuring that the team had access to all contact details, and avoiding or reducing discussion on topics that might cue distress:

*“Sessions tended to be client led in terms of lack of intensity*, *but therapists worked to contain risk*. *There was possibly more liaison with other services to manage risk and discussion about highly distressing material was contained*.*”*^*3–47*^

*“brainstorming how to manage various TIB such as* … *’walk outs’”*^1–84^

*“Developing safe operating procedures and practices around using technology and new ways to manage risks and escalate concerns*.*”*^3–104^

#### Privacy concerns

Privacy was mentioned frequently both in relation to individual and skills training. These included concerns about the security of online platforms, and risk of group members breaching each other’s confidentiality when joining group from non-private settings.

Respondents described the following solutions: conducting research on the merits and security of various platforms, advocating with their leadership to support the use of telehealth and/or a particular platform despite privacy threats, developing information sheets and agreement forms regarding the limits and risks of telehealth, and encouraging clients to use headsets.

*“checking the facts re*. *platform privacy*, *seeking executive support for use of platform and acknowledgement of organisational risks”*^1–1^

*“With the assistance of some more technologically-able clients*, *we explored all provider services for ease of use and clarity of connection*. *In the best interests of our already highly-stressed clients*, *we resolved to stick with Zoom in spite of concerns from management*. *We created a new Consent Form for clients to sign describing the risk of doing therapy from teleconference*.*”*^1–66^

*“various virtual platforms were utilised (eg*: *zoom where coviu was unsustainable); rearranging appointment times based on privacy and availability; clients finding a safe space (eg*: *car or park) to link into the session where privacy wasn’t achievable at home; telephone sessions where telehealth wasn’t possible*.*”*^1–59^

#### Logistical barriers due to virtual rather than in-person connection

Respondents noted difficulties with distributing materials, not having shared access to a physical whiteboard, and challenges with engaging in experiential skills practice, such as mindfulness exercises or role plays in remote individual and group sessions. Completing chain analyses was harder remotely, and electronic materials had to be fillable online, as clients and clinicians lacked access to scanners and printers at home.

*“Teaching issues—especially complicated use of experiential exercises—we had relued[sic] quite heavily on small group exercises/role plays”* ^3–68^

*“all resources outside powerpoint slides were paperbased (questionniares*, *workbooks*, *exercises)—difficult to get resources to people due to postal delays at the time*, *and return rate of questionnaires was much lower*.^2-71^

*“Group discussion and small group practice is harder*. *Difficult to share content easily while also teaching*. *Lots of need to email materials in advance = admin burden*.*”*^2–31^

Solutions involved sending out materials in advance, selecting online-friendly activities, collaboratively searching for solutions, and engaging in more preparation and orientation activities before the session by phone, text or within-sessions.

*“Used the phone voice calling and txt to help clients sort out tech issues prior to and during group*. … *Invited clients into discussion instead of waiting for input*, *talked about difficulties*, *used powerpoints on share screen to explain eg model of emotions etc organised hard copies for collection*, *snail mailed*.*”*^3–47^

“*We both mailed and emailed copies of the week’s handouts prior to the session*.”^1–66^

*“Brainstorming and research on ideas to increase engagement as well as practicing and trying out mindfulness and teaching strategies in Consult Meetings*.*”*^2–3^

#### Managing therapy interfering behaviour

Challenges with managing therapy interfering behaviour from clients via telehealth were mentioned frequently. Concerns related to client visibility, which was most frequently attributed to unwillingness to be seen on screen, but sometimes to data or connectivity limitations. Respondents also reported a greater frequency of non-attendance, and eventual dropouts.

*“Clients can switch off their video*, *leaving therapists unaware of why / what is happening in their location*. *disruption to audio / video and internet connection can be very stressful in Zoom groups and hard to troubleshoot while clients are anxious / needing reassurance*. *Zoom chat is not effective means for checking in and supporting distressed group members*.*”*^2–31^

*“Transition to teleconferencing led a spike in dropouts*.*”*^3–68^

“*therapy interfering behaviours much harder to assess and address*”^1–108^

Solutions to this included the development of telehealth guidelines, using more engagement strategies and reminders, and for some programs, particularly those running hybrid groups, having an additional facilitator i.e. one to manage in-person group members, and another to provide phone or chat coaching for participants attending remotely.

*“worked extra hard at engagement*. *Used email a lot more for phone coaching*, *relationship repair and cheerleading -worked with clients around these and treated as therapy interfering behaviours*”^1–72^

*“Most importantly*, *we set up guidelines for "Doing Virtual DBT"*. *These were rules about the etiquette of teleconferencing and how to prepare for the session…Therapists and clients became more confident and relaxed*. *Even though spontaneous interactions were curtailed*, *the more structured process was helpful for teaching the material*”^1–66^

*“clear group guidelines and expectations set for sessions and revisited as and when needed (addressed as a TIB in individual as well); extra facilitator as a "skills coach" for the first couple of modules that could take someone into a break out room when they were distressed—to allow facilitators to keep teaching skills*.*”*^1–59^

### Perceived barriers for therapists and clients

#### Therapeutic affiliation/connection

Respondents noted concerns about the impact of telehealth on a sense of connection and subsequent engagement, affecting both the therapeutic relationship, and for group, the sense of affiliation with other group members.

*“It is difficult to engage young people over video-conference*, *harder to connect virtually*”^1–41^

*“A number of clinicians reflected the loss of richness in therapeutic connection over Zoom*.*”*^1–60^

*“Challenging to get clients to interact with each other and feel supported (loss of group interaction and feeling of support and cohesion)*.*”*^2–3^

Solutions included creating opportunities for more socialising in groups, and increasing therapist animation and warmth.

*“Used zoom chat room function with facilitators dropping into rooms to oversee*. *Not as efficient or engaging as face to face seemingly but definitely useful*. *Managing group connectedness was challenging—we provided space to discuss this openly and grieve this lost opportunity*.*”*^3–68^ *“additional group activities to provide opportunities for connection*, *facilitators being more animated when presenting…Attempted to allow additional time to talk at the start of group for networking”*^1–76^

#### Lack of willingness

Many respondents mentioned clients and therapists being unwilling to engage in telehealth as a significant barrier to using this method of delivery. This seemed to be driven both by lack of confidence in its efficacy as well as simply finding it aversive, and insufficiently reinforcing to do. Descriptions of staff unwillingness included terms such as preference.

*“staff reluctance to use telehealth as a platform to deliver health services*.*”*^3–104^

*“I also felt I really didn’t want to shift to Telehealth*, *as a personal preference*.*”*^2–48^

Descriptions of client unwillingness were more likely to be framed in terms of avoidance or lack of motivation.

*“clients anxiety leading to avoidance behaviour*, *some disliked telehealth*”^1–50^

*“More difficulty engaging via Zoom*, *seeming to be due to motivation and disliking the experience of Telehealth*”^1–82^

*“Client reluctance to participate in group sessions via telehealth—including the belief DBT skills training would ’not be the same’ if not in person/in the same room as other participants*.*”*^2–23^

Respondents mentioned consult team as a source of solutions for lack of willingness, modelling and encouraging clients and fellow-therapists to give telehealth a try, and also highlighting the freedom to choose, in circumstances with few options. Validation of the difficulty of adapting to remote therapy was mentioned frequently, as was various permutations of the foot-in-the-door DBT commitment strategy, including a couple of programs having administrative staff involved in the process of encouraging and helping clients to give telehealth a try.

*“Consult team to identify alternatives (e*.*g*. *option of home visits—also had barriers)*, *then skills to increase therapist willingness in absence of alternatives*”^1–1^

*“Identified those within the team that were willing to try something new and demonstrate to other team members how well it could work by sharing their practical tips and knowledge*.*”*^3–104^

*“Options were given to*:*—try it for a session—have reception assist with tech set up and practice prior to session—validation for concerns and psycho education about effectiveness and/ other client’s feedback on video was surprising similar*”^2–71^

*“commitment strategies to increase willingness to use zoom problem-solving/radical acceptance Used Walking Middle Path Skills and commitment strategies*.*”*^1–82^

*“Staff member contacting individual clinets[sic] and supporting them in problem solving and trying online- foot in the door worked well for these- once they came once were happy to continue*”^1–44^

Other barriers were not specific to the delivery of DBT per se, but rather related to lack of technical knowledge and confidence, access to space, and lack of necessary resources (devices, data, information systems and platforms) to provide telehealth. Respondents noted these problems being relevant to both therapists and clients. For the most part, solutions involved getting access to the requisite information and resources.

#### Lack of technical knowledge

Lack of confidence was mentioned frequently with this theme: therapists having a lack of confidence in their ability to use technology, in the efficacy of therapy via telehealth, and in the adaptation of skills content. Concerns often centred around the threat to the quality of therapy due to this barrier.

*“Most of our therapists were unfamiliar with teleconferencing and did not manage the groups very effectively*. *Clients and therapists found it stressful*, *discouraging and de-motivating*”^1–66^

*“Some also at the beginning of Covid restrictions also lacked the confidence in their technological competency to run individual therapy via telehealth*, *let alone group therapy*.*”*^2–35^

*“Initial teething problems while clients and clinicians became familiar with the technology*.*”*^1–106^

*“Some clients struggling to use the technology efficiently*.*”*^2–31^

The critical role of effective tech support was frequently mentioned as a solution. Other solutions for staff included in-services, training, and cheat sheets, and the role of telehealth ‘champions’ within services who trialled and troubleshot the use of telehealth, and then modelled solutions to technical challenges for other team members. Similarly, solutions for clients involved coaching, modelling and training.

*“Education and training*. *Fact and tip sheets… Identified those within the team that were willing to try something new and demonstrate to other team members how well it could work by sharing their practical tips and knowledge*.*”*^3–104^

*“Staff inservice on running online groups*. *Staff practiced strategies with each other*.*”*^2–3^

*“Some staff eventually educated themselves re technical issues and IT department provided some education*, *although this was limited*. *Over time clients and staff became a little more confident re using Telehealth*.*”*^4–51^

*“trouble-shooting and support offered for learning how to engage in the group via zoom—a document was created on DBT group via zoom with etiquette and practical information and provided to all group members and prospective members*”^1–36^

#### Lack of private spaces to do DBT via telehealth

Participants described both clients and therapists struggling with issues of space, especially in crowded worksites and homes, and when children, flatmates and other family members were also at home. In particular, absence of private spaces raised issues for clients who were at risk of domestic violence.

*“Lack of space (crowded offices with multiple phone calls happening)*”^1–66^

Solutions focussed on the creative use of private spaces, including staff and clients accessing virtual platforms from vehicles, clinics highlighting the need to use headsets, and the provision of (empty) clinic ‘zoom rooms’ for clients who didn’t have private spaces at home to access telehealth.

*“Wireless head sets for use with phones Creative use of available spaces (eg conducting phone session in photocopy room)*.*”*^1–67^

*“Assisted participants to problem solve (e*.*g*., *some sat in their car while having a session if no safe place inside home)*”^4–24^

#### Lack of resources

For staff, this barrier included challenges with having organisational approval for the use of platforms, delays in accessing equipment, trouble obtaining professional remote platform software that allowed multiple log-ins, and ensuring all DBT therapists had access to computers while working remotely. This was complicated by organisations vetoing the platforms that were most accessible for clients. The primary solution involved the organisation providing access to resources. However, a number of participants reported staff used their own personal equipment and platform accounts to make telehealth viable.

*“A staff member used their personal paid account at Zoom initially as no other options*. *Then service banned Zoom and insisted on Teams but this was then worse but remains poor for utility in running groups and being user friendly and accessible for clients*. *We didn’t have clients emails*. *Accessing secure internet and online facilities (laptops/ cameras/ mics etc)*”^1–44^

*“Not all clinicians could access the requirements for VC calls- camera*, *microphone*, *private space… Service being very particular about what VC platforms were allowed and clinicians and clients having access to these*.*”*^1–44^

*“Therapists were provided with technology upgrades*, *eg*, *more data allowance*, *dual monitors and webcams*. *we also had private spaces*.*”*^1–66^

Sometimes the challenges were exacerbated by lack of reimbursement for telehealth sessions

*“Funding model—majority of our patients are funded through private health insurance*. *It took approx*. *2 months for private health insurers to agree to approve telehealth sessions (despite Medicare and other COVID specific approvals being in place) and even then with considerable restrictions*.*”*^4–24^

Respondents noted disparities between resources they as therapists could access, and those available to their clients. Client data insecurity was mentioned particularly often.

*“clients were less well-resourced—unreliable connectivity*, *insufficient data allowance old equipment etc*. *Nor did they necessarily have private uninterrupted spaces available in their homes*.*”*^1–66^

*“Clients who were using their phones used up their data quickly*, *and tended to have less effective connection than those using computers*.*”*^1–106^

Solutions included the provision of equipment for clients who lacked this, facilitating access to software and data, and actively problem-solving challenges if the service was unable to intervene directly to meet the need.

*“Continued to push [management] to get funding arrangement in place*”^4–24^

*“-our [service] loaned dongles for wifi and tablets to clients*”^1–72^

*“problem solving with client*, *providing information re*. *free internet upgrades*”^1–1^

*“Liaised with patients to offer as many options as possible (e*.*g*., *participating using phone screen*, *tablet*, *etc) & posting out hardcopy handouts if not printer available*”^4–24^

#### Barriers reported by team leaders for Indigenous and Pacific People clients

Only eight respondents endorsed specific barriers for Indigenous and Pacific People clients. Team leaders endorsing this item described problems accessing devices, data and private spaces, juggling competing demands related to childcare and a preference for face-to-face sessions.

*“No private space at home Limited internet No laptop / tablet These barriers are experienced by many in our community due to low incomes and inadequate housing*”^1–67^

*“Access to technology /wifi*. *Having a private and quiet space (lots of over crowding in peoples homes)*. *Limited Phone credit*.*”*^3–104^

## Discussion

The current study focussed on understanding obstacles to providing individual DBT and skills training via telehealth and solutions to those barriers, from the perspective of DBT team leaders in Australia and New Zealand. Respondents were based within a range of health services, treatment settings, and service user populations. The majority of survey respondents (77%) reported their teams had provided at least one DBT mode via telehealth. It is noteworthy that the majority of teams found a way to transition to telehealth without any official guidance. This may represent the common commitment by Australian and New Zealand clinicians to ensure their DBT clients received services during a particularly stressful time for both service users and therapists. Importantly, not all teams moved to telehealth. The most frequently mentioned reasons for this involved teams being prohibited from doing so by organisation managers. That said, other reasons indicated that teams simply were not ‘telehealth-ready’. The early days of lockdowns were highly stressful for health professionals. Arguably, while many or most services were not telehealth ready, there may have been different levels of readiness which may have impacted transitioning.

Qualitative analysis of responses revealed two broad and overlapping categories of perceived barriers for therapists and for clients, with concerns about access to necessary resources (software, hardware, and technical knowledge and skill) contrasting with concerns about process (including concerns about therapeutic connection, managing risk and dysregulated behaviour, and lack of confidence in this medium), seen as common to both groups. For Indigenous and Pacific People clients who were unable to engage in telehealth, the most frequently reported barrier by team leaders was lack of access to resources and privacy, however this finding has to be viewed with high caution as it is based on such a small number of respondents.

Problems with engagement and alliance were a recurrent theme in both anticipated barriers for respondents whose teams did not transition, and perceived barriers for those leaders whose teams did transition to DBT delivery via telehealth. This echoes the finding from a pre-pandemic comparison of online versus in-person DBT skills groups that while attendance was better for online groups, cohesion with other group members was lower [23].

Multiple respondents identified barriers that were more related to systemic issues (organisations not allowing telehealth, the challenges with the provision of hardware and accessible platforms to deliver telehealth, adequate training and support) rather than issues specific to clients or staff. As such, these findings build on the information reported by Zalewski and colleagues, whose research focussed on barriers and solutions at the level of therapists, and included a brief mention of the role of organisational advocacy [9]. It underscores the importance of organisational support and infrastructure in delivering DBT via telehealth noted by Landes and colleagues [13]. Findings from the current study have significant implications for the critical role of managers at all levels to support the delivery of DBT via telehealth if implementation efforts are to be successful.

Solutions identified by respondents to the above barriers focussed on: actively advocating and problem-solving access to necessary resources for clients and clinicians, compensating for or recouping the perceived disadvantages of telehealth, using DBT skills and strategies to solve problems, developing shared expectations and processes for clients and therapists, and increasing the digital skills of the DBT team and the clients receiving treatment from that team. Respondents’ comments repeatedly highlighted the pivotal role their organisation held in the delivery of remote DBT, from the most basic step of actually allowing DBT via telehealth to occur, through to the provision of software, hardware, connectivity, training and private space integral to its delivery. Importantly, for many DBT services, the successful transition to telehealth required that these resources be provided for clients, as much as therapists. This is an essential consideration if services are to avoid widening inequities due to digital exclusion [24].

These findings underscore the need for services to factor in the costs of providing/lending devices, and covering data expenses when planning telehealth delivery and to have a strategy for facilitating access to private spaces for service users who do not have one. This is particularly relevant to clients experiencing overcrowding, and homelessness.

The finding that the most frequent barrier to participation was lack of needed resources highlights an important source of inequity. Equipment access and data insecurity were frequently mentioned by respondents as general barriers to telehealth DBT. At first glance, there may be an assumption that telehealth would improve treatment access for patients in remote locations. However if living in a remote location co-occurs with digital exclusion due to lack of resources or knowledge [24], then telehealth will increase the health gap rather than reducing it. There is a risk that this may particularly impact Indigenous and Pacific People service users given the social and economic disparities for these ethnic groups in Australia and New Zealand.

These findings tally with previous research describing the challenges and solutions reported in the lessons learned by DBT clinicians from predominantly North American samples who transitioned to telehealth over a fairly similar time period [9]. It is striking that services on opposing hemispheres generated similar solutions to these common problems, and may speak to the power of digital communication to span physical distance through the use of platforms such as practitioner listservs. It is also noteworthy that such a high proportion of respondents in the current sample transitioned to video conferencing in some form. It is possible that the range and flexibility of solutions employed to make this shift may speak to the role of both DBT skills and DBT consultation teams to support creative and nimble responses to novel problems.

This study adds to the growing pool of information about the remote delivery of DBT, an area that holds high relevance for the field. The strengths of this study include the focus on a particular geographic region of the world where the healthcare services are relatively homogenous; focus on specific modes of DBT, the inclusion of leaders of teams who did not transition to remote delivery of DBT and the attempt to explore perceived barriers experienced by Indigenous and Pacific People clients who were seen as unable to engage in DBT telehealth.

This study has a number of limitations. In particular, it is based solely on the perceptions of leaders of teams, rather than DBT clinicians more broadly, and does not include the views of DBT service users. Accordingly, it only reveals the perspectives of a small group, and this is a significant constraint on the validity of the findings. DBT team leaders are more likely to have represented the views and experience of their fellow therapists, however it is highly likely that service users will have different perspectives on DBT via telehealth. This is an important gap in our current knowledge of DBT telehealth delivery and a necessary focus for future research. That said, the perspectives of DBT team leaders are likely to represent the experience of their team members, and yield important information about barriers and solutions to the delivery of DBT via telehealth. While the proportion of the sample who transitioned to telehealth may not accurately represent the proportion of all teams in Australia and New Zealand that made this shift, the participants’ qualitative observations still hold meaning.

It’s unclear how much the respondents’ views represent what occurred with DBT programs in Australia and New Zealand during lockdown. There is no common register of DBT programs in Australia or New Zealand and hence it’s not possible to determine the proportion of program leaders who responded from the total number of functioning programs in these countries. The majority of respondents led teams who did transition to DBT via telehealth during lockdown. It is unclear whether there was a response bias, such that teams who had transitioned to delivering DBT via telehealth were more likely to respond. Hence, the current data are unlikely to capture fully the experiences of teams who did not transition to DBT via telehealth. There could well be some insurmountable barriers that are not identified here that are highly relevant to future endeavours to support teams to move towards DBT via telehealth. That said, over 40% of respondents indicated that they did **not** offer DBT via telehealth for one modality or other, and thirteen team leaders indicated that their programs did not deliver either modality via telehealth during lockdown at all. Therefore this study has important information from a sizable minority regarding barriers to the delivery of DBT via telehealth in Australia and New Zealand.

A further significant limitation relates to the data from respondents commenting on the experience of Indigenous and Pacific People clients. First, only eight respondents identified specific barriers for these groups, significantly limiting the generalisability of these findings. Second, the design and language used for items enquiring about barriers for Indigenous and Pacific People clients may have skewed responses. Specifically, asking whether individuals were *unable* to continue DBT via telehealth may have primed respondents to attribute a lack of participation in telehealth to an inability to take part. Respondents who perceived clients as being able to participate may not have endorsed the item, and therefore their perceptions of the factors associated with those individuals would not be included in this analysis. In hindsight it would have been more effective simply to ask team leaders to comment on their perceptions of the reasons that clients did not continue with DBT telehealth. Further work is sorely needed to understand the specific barriers experienced by Indigenous and Pacific People service users of DBT via telehealth. The current paper’s efforts to do so highlights that need, and illuminates the changes required to methodology to improve the assessment of access inequities.

This study also offers a series of tailored solutions to commonly-reported barriers that can inform the delivery of remote DBT for DBT teams and their organisations when planning future telehealth initiatives. In addition, it highlights a number of actions that implementation and dissemination experts could undertake to prepare the field for telehealth delivery. Over the past decade a number of authors have embedded links in their DBT treatment manuals to digital copies of the handouts and worksheets associated with their adaptation. Digital access to materials is particularly important in telehealth, in terms of both reducing the burden on clinicians, and developing frictionless processes for getting materials to clients. At least one DBT treatment developer has made fillable pdfs of some of her worksheets available in the public domain [25]. Having a shared commitment in the DBT treatment development community to create access to fillable versions of electronic materials would be very useful. Revising the agreements under which previous texts have offered digital access to materials, to allow electronic (and where relevant fillable) versions of handouts and worksheets for therapists to provide directly to clients is urgently needed in practice. However, this is only relevant to the extent that service users have access to devices that allow electronic completion. To this end, having mobile phone-friendly versions of the standard DBT worksheets is particularly important, both for engaging in therapy and also enhancing skills generalisation.

An important next step in future research on DBT telehealth is to gather quantitative data on outcomes associated with this mode of delivery. This would complement the information we have on the challenges and solutions identified by DBT practitioners to telehealth implementation. A further line of enquiry is DBT consumers’ experience of telehealth, particularly from those who dropped out of DBT during the period(s) this was offered. Having more information about the factors that led to individuals withdrawing from treatment delivered via telehealth will also identify the barriers to prioritise addressing.

## Conclusions

The results of this study add further support to the growing evidence base indicating that DBT is feasible to implement via telehealth and has the potential to enhance its reach. Importantly, the shift of DBT to online platforms relies on organisational support to provide good telehealth, including the provision of equipment, a secure and accessible platform, reliable connectivity, and effective technical support to clinicians. However, ensuring that clinicians have access to the resources needed to extend DBT via telehealth is meaningless if service users lack the corresponding resources to receive it. Organisations must include plans for resourcing consumers’ access to those platforms, otherwise DBT via telehealth is only an option for those with the devices and data to access it.

## Supporting information

S1 File (PDF)

S2 File (PDF)
