# Supplementary material for: Getting DBT online down under: The experience of Australian and New Zealand Dialectical Behaviour Therapy programmes during the Covid-19 pandemic
Source: PLoS One. 2022 Oct 6;17(10):e0275636. doi: 10.1371/journal.pone.0275636 (PMC9536633; doi:10.1371/journal.pone.0275636)
Supplement: S1 File — (PDF) [file pone.0275636.s001.pdf]

CODEBOOK

Perceived barriers for therapists

Assessment & management of emotions and high-risk behaviours, particularly behaviours generating concerns about safety
Privacy and information security concerns
Logistical challenges related to non-physical meetings
Managing therapy-interfering behaviour is harder via remote platforms

Perceived barriers for therapists and clients

Disruptions to therapeutic alliance
Lack of willingness
Lack of technical knowledge
Lack of private spaces to do DBT via telehealth
Lack of resources

Solutions to perceived barriers for therapists

Development of telehealth safety protocols
More liaison and communication by clinicians regarding suicide and self-harm risks with service user and others
Confirmation that service has access to all clients' contact details
Clinicians may avoid distressing topics if the client seems already distressed
Gather information on security of various video call platforms
Advocacy by managers and clinicians to use telehealth despite privacy concerns to improve access for clients
Development and distribution of information sheets and agreement forms on limits and risks of telehealth for clients
Mailing copies of handouts and worksheets ahead of time
Arrange for clients to email completed materials ahead of therapy, or or hold completed materials up to camera
Tailor activities to online format in choice of exercises and use of media
Inform clients what to bring to session
Set more time aside for pre-session preparation and orientation activities for clients
Create guidelines describing expectations of therapy via telehealth
Use more engagement strategies and send out more email/text reminders to maintain engagement
Target problem behaviour in individual and skills sessions
Option of including an additional facilitator in telehealth group skills training sessions to manage clients' therapy-interfering behaviours
Use breakout rooms to coach individuals
Clinicians to initiate more frequent contact between sessions

Solutions to perceived barriers for therapists and clients

Acknowledge and discuss the problems of disruptions to alliance with clients
Clinicians to validate and increase use of phone, email and text to improve therapeutic alliance
Clinicians can encourage more chat and socialising in groups during group breaks
Clinicians to be more animated and exude more warmth during individual therapy and group sessions
Clinicians to encourage group members to reach out to each other during breaks
Clinicians can request consent for clients to share their completed homework sheets with the group

DBT teams can problem-solve in consult meetings, and explore reasons for unwillingness amongst staff and clients
Clinicians can role-model giving telehealth a try and invite fellow team members and clients to do the same
Teams can highlight freedom to choose in the absence of desirable alternatives to DBT telehealth, to both clients and fellow DBT team members
Service can validate staff and clients struggles
Service can provide information about others' positive experience of telehealth
Service can resource administrative staff to encourage clients to schedule a first telehealth session
Service to increase tech support to both clients and therapists
Service or managers to educate and train staff in telehealth and the video call program of choice
Identify and support telehealth clinician 'champions' who trial, troubleshoot and model solutions, and then feedback to the rest of the team
Clinicians to orient and coach clients
Development of tip sheets for clinicians and clients
Encourage use of headphones for clinicians and clients
Increase access of individual devices and spaces for clinicians where possible, improve resources for clinicians from a service-level
Actively problem-solve and use flexibility re location of therapy sessions for privacy (e.g. cars) if no other private spaces are available
Provide the option of private rooms in GP or other community service space for clients
Managers and clinicians to advocate for provision of software, hardware and connectivity for clinicians and clients
Use ethernet rather than wifi to improve internet connection
