# Supplementary material for: Getting DBT online down under: The experience of Australian and New Zealand Dialectical Behaviour Therapy programmes during the Covid-19 pandemic
Source: PLoS One. 2022 Oct 6;17(10):e0275636. doi: 10.1371/journal.pone.0275636 (PMC9536633; doi:10.1371/journal.pone.0275636)
Supplement: S2 File — (PDF) [file pone.0275636.s002.pdf]

BARRIERS

What barriers did your centre encounter to providing individual therapy via telehealth during COVID-19's lockdown period?

Access to stable internet connection for use of Zoom / telehealth platform. Some clients
struggling to use the technology efficiently. Clients having difficulty ensuring a private and
distraction-free environment to undertake telehealth. Some difficulty assessing affect and
2-31 personal risk / safety in telehealth.

It is difficult to engage young people over video-conference, harder to connect virtually, difficult
for young people who use avoidance as a coping strategy, more difficult to engage in chain
analysis due to technology (So without whiteboards in rooms etc), difficult given we engage both
young people and their parents in the program so the young person didn't get as much time on
their own due to family being at home (unable to have a safe space outside of the home), there
were initial concerns around the management of risk (unable to complete risk assessments in
person or at the clinic, if people dropped offline if was more difficult to manage initially then if
1-41 someone walks out in a group - you can follow up if they don't return)

Not all clinicians could access the requirements for VC calls- camera, microphone, private space.
Telephone calls were problematic on many levels and many clients didn't like them. General
service demands to respond for all clients to pandemic made it hard to be as reliable in timing and
give full hour to IT sessions. Clinicians not having skills or experience in use of VC technology
clinically. Service being very particular about what VC platforms were allowed and clinicians and
1-44 clients having access to these.

remote access to ...computer system, clients anxiety leading to avoidance behaviour, some
1-50 disliked telehealth

Some clients found it difficult to utilise Zoom. A number of clinicians reflected the loss of richness
1-60 in therapeutic connection over Zoom.

Lack of space (crowded offices with multiple phone calls happening). Clients feeling isolated at
1-66 home

More difficulty engaging via Zoom, seeming to be due to motivation and disliking the experience of
1-82 Telehealth, though some participants really appreciated it.

Some clients not interested in zoom. Difficult to get any therapeutic traction over zoom. Losing
1-99 more subtle interpersonal interactions. Issues with technology.

staff reluctance to use telehealth as a platform to deliver health services. Developing safe
operating procedures and practices around using technology and new ways to manage risks and
3-104 escalate concerns.

Initial teething problems while clients and clinicians became familiar with the technology.
Inconsistent privacy at home to be able to participate in telehealth. Clients who were using their
phones used up their data quickly, and tended to have less effective connection than those using
1-106 computers. Technical issues with sound/vision at times, ate into session hour.

What were the barriers to your centre providing group skills training via telehealth during COVID-19's lockdown period?

No Medicare rebate was available for group Telehealth, most participants use the rebate. Also did
not feel there was any evidence base to support telehealth delivery of skills trading and I looked
for research to support it. Felt it may be ineffective and reflect poorly on my practice. I also felt I
2-48 really didn't want to shift to Telehealth, as a personal preference.

What barriers did your centre encounter to providing group skills training via telehealth during COVID-19's lockdown period?

Identifying a secure and reliable platform for telehealth. Clinician confidence in managing the
technology. Clinician confidence in engaging with clients in a group online and effectively
providing skills training. Some clients reluctant to participate in the group online or spending
2-3 many hours for work online and unwilling to spend more hours in group online. Challenging to get

clients to interact with each other and feel supported (loss of group interaction and feeling of support and cohesion). Clients struggling with maintaining focus and issues with dissociation. NO MEDICARE REBATES AVAILABLE FOR TELEHEALTH GROUP SESSIONS FACILITATED BY PSYCHOLOGISTS/SOCIAL WORKERS. I have capitalised this statement on purpose. I note that Medicare rebates for telehealth group sessions were available for psychiatrists. I am not aware of any private practices, public hospitals or private hospitals at which psychiatrists facilitate group sessions. This provision of Medicare rebates for telehealth groups looks genuine 'on paper', but anyone who works in the mental health system knows it is an empty provision - there will be no uptake. Client reluctance to participate in group sessions via telehealth - including the belief DBT
2-23 skills training would 'not be the same' if not in person/in the same room as other participants.

1) Funding model - majority of our patients are funded through private health insurance. It took approx. 2 months for private health insurers to agree to approve telehealth sessions (despite Medicare and other COVID specific approvals being in place) and even then with considerable restrictions. 2) Patients being able to access technical equipment if they did not already have a laptop and other equipment 3) Therapists not being experienced/confident about adaptation of group skills content 4) Concerns around risk management when interacting over telehealth rather than face to face 5) Some patients not wanting to engage in telehealth due to confidentiality
4-24 concerns (e.g., no safe or appropriate place at home to be able to participate in an online group)

Assessing group engagement and monitoring participants affect / coping in sessions is difficult when you can't see everyone on the same screen. Conversation can become stilted in groups via telehealth when people interrupt. Background noises are less controllable and more distracting. Clients can switch off their video, leaving therapists unaware of why / what is happening in their location. disruption to audio / video and internet connection can be very stressful in Zoom groups and hard to troubleshoot while clients are anxious / needing reassurance. Zoom chat is not effective means for checking in and supporting distressed group members. Group discussion and small group practice is harder. Difficult to share content easily while also teaching. Lots of need to
2-31 email materials in advance = admin burden.

A staff member used their personal paid account at Zoom initially as no other options. Then service banned Zoom and insisted on Teams but this was then worse but remains poor for utility in running groups and being user friendly and accessible for clients. We didn't have clients emails. Accessing secure internet and online facilities (laptops/ cameras/ mics etc) plus having experience in adapting the group material etc to online delivery format. Clients refusing to move online/
1-44 saying couldn't.

Therapists were provided with technology upgrades, eg, more data allowance, dual monitors and webcams. we also had private spaces. However, clients were less well-resourced - unreliable connectivity, insufficient data allowance old equipment etc. Nor did they necessarily have private uninterrupted spaces available in their homes. Most of our therapists were unfamiliar with teleconferencing and did not manage the groups very effectively. Clients and therapists found it
1-66 stressful, discouraging and de-motivating

Transition to teleconferencing led a spike in dropouts. Hard to know why as those who dropped out were difficult to contact. one did not have adequate privacy at home. Group affiliation seemed to suffer and several lamented that they missed the close human contact from face to face group. Teaching issues - especially complicated use of experiential exercises - we had relued
3-68 quite heavily on small group exercises/role plays

- Changes in the COVID rules for groups. - Started as f2f then moved to all Zoom (with 2 days notice) - faciliatator preferred f2f format, opted to not run future Zoom groups. - needed to train up a new facilitator to run Zoom group. - rapid move to Zoom didn't leave enough time to do adequate training on tech for faciliatator and co-facilitator, and group participants - no Medicare items for group initially - all resources outside powerpoint slides were paperbased (questionniares, workbooks, exercises) - difficult to get resources to people due to postal delays at
2-71 the time, and return rate of questionnaires was much lower. - We needed to move everything to

having digital access including consent forms as both clinicians and clients were working from home and neither had access to scanners/ faxes to return forms etc.   - processes were based on group members coming in to the practice. Needed to incorporate additional safety checks e.g., primary and secondary location of Zoom participants.   - participants needed more breaks in the group. - group (telehealth) guidelines were needed so that participants didn't eat, leave computer etc.   - we did a hybrid format - some on telehealth, some f2f (limit of 5 people on room and all wearing masks). For this we needed an additional co-faciliatator to do phone/ chat coaching during group - if someone left computer; and another co-facilitator in the group room should a participant leave the room. - various tech problems like the reminder with Zoom link included a full stop - which meant that participants who copied and pasted it didn't get into the Zoom room, whereas those who clicked on the link did. These problems were hard to diagnose.   - needed additional tech support. IT required to be available during running of group.

What were the barriers to your centre providing group skills training via telehealth during COVID-19's lockdown period?

Our DBT team were not confident they would be able to provide the same level of duty of care to clients via telehealth as in a face to face group.   Some also at the beginning of Covid restrictions also lacked the confidence in their technological competency to run individual therapy via telehealth, let alone group therapy. However a study ... showed this quickly changed with regards to individual therapy.   Our DBT Team also felt a large part of the DBT model is the opportunity for participants to get to know each other over refreshments etc and this feel would not be possible
2-35 via telehealth.

SOLUTIONS

What solutions did you arrive at to overcome these barriers? Individual

Consult team to identify alternatives (e.g. option of home visits - also had barriers), then skills to increase therapist willingness in absence of alternatives, checking the facts re. platform privacy, seeking executive support for use of platform and acknowledgement of organisational risks,
1-1 problem solving with client, providing information re. free internet upgrades

1-82 commitment strategies to increase willingness to use zoom   problem-solving/radical acceptance

Tried to help clients sort out tech issues prior to and during sessions. Requested use of headsets, discussed confidentiality issues with clients, troubleshooted difficulties. Sessions tended to be client led in terms of lack of intensity, but therapists worked to contain risk. There was possibly more liaison with other services to manage risk and discussion about highly distressing material
3-47 was contained.

Some clients were seen in person (with PPE and distancing) where clinically appropriate; various virtual platforms were utilised (eg: zoom where coviu was unsustainable); rearranging appointment times based on privacy and availability; clients finding a safe space (eg: car or park) to link into the session where privacy wasn't achievable at home; telephone sessions where
1-59 telehealth wasn't possible.

With the assistance of some more technologically-able clients, we explored all provider services for ease of use and clarity of connection. In the best interests of our already highly-stressed clients, we resolved to stick with Zoom in spite of concerns from management.   We created a new
1-66 Consent Form for clients to sign describing the risk of doing therapy from teleconference.

Wireless head sets for use with phones Creative use of available spaces (eg conducting phone
1-67 session in photocopy room). Using postal service to mail hard copy materials to clients

Options were given to: - try it for a session - have reception assist with tech set up and practice
2-71 prior to session - validation for concerns and psycho education about effectiveness and/ other

1-72	client's feedback on video was surprising similar - let them know that we would prioritise them for the option to return to f2f as soon as was safe to do so - mask was offered as an alternative -worked extra hard at engagement. Used email a lot more for phone coaching, relationship repair and cheerleading -worked with clients around these and treated as therapy interfering behaviours
1-76	Attempted to allow additional time to talk at the start of group for networking, attempted to acknowledge and problem solve impacts of telehealth appointments
1-84	very quick study of tech and legal issues arising. Making agreement forms for clients to help ensure each other's privacy. ensuring contact phone details. online practice sessions. brainstorming how to manage various TIB such as LTB or 'walk outs'
3-104	Education and training. Fact and tip sheets. Identified those within the team that were willing to try something new and demonstrate to other team members how well it could work by sharing their practical tips and knowledge. Deliver in house training and support. Create procedures around risk management and safe telehealth practices.
	**What solutions did you arrive at to overcome these barriers? Group**
2-3	Research in to platforms that could meet the needs of a skills training session. Staff inservice on running online groups. Staff practiced strategies with each other. Validation of clients concerns re time spent on zoom with work and study and some clients chose to withdraw. Brainstorming and research on ideas to increase engagement as well as practicing and trying out mindfulness and teaching strategies in Consult Meetings.
1-108	Lots of commitment strategies to increase clients' uptake of telehealth groups. Most of our clients continued to attend sessions despite the lack of [insurance] rebates - the rebates for group sessions and the number (10 per calendar year) is completely inadequate anyway, so most had utilised all of their group rebates prior to COVID anyway.
4-24	1) Continued to push ... to get funding arrangement in place (the DBT program was the only one ... to end up delivering telehealth) 2) Liaised with patients to offer as many options as possible (e.g., participating using phone screen, tablet, etc) & posting out hardcopy handouts if not printer available 3) Adapted content to online setting where possible; consulted with clinicians in other spaces... to find novel ways to present material 4) Created new procedure for risk assessment and management over telehealth 5) Assisted participants to problem solve (e.g., some sat in their car while having a session if no safe place inside home) and others chose not to participate in telehealth but re-enrolled in the group when we returned to face to face
1-36	- some people were chose not to continue on with/join a group because it was online - trouble-shooting and support offered for learning how to engage in the group via zoom - a document was created on DBT group via zoom with etiquette and practical information and provided to all group members and prospective members
1-44	Lots of hours of a staff member trouble shooting the platforms and training herself to use them then training other staff and clients; developing info sheets for clients; Staff member contacting individual clinets and supporting them in problem solving and trying online- foot in the door worked well for these- once they came once were happy to continue; adapting content for new delivery modality; heading the groups by ensuring had access and leading the group in adjusting together.
3-47	Used the phone voice calling and txt to help clients sort out tech issues prior to and during group. Developed a confidentiality agreement, required use of headsets, discussed confidentiality issues with each client individually prior to group and troubleshooted difficulties. Invited clients into discussion instead of waiting for input, talked about difficulties, used powerpoints on share screen to explain eg model of emotions etc organised hard copies for collection, snail mailed.
4-51	Eventually the institution bought (some) equipment & plugging into data sockets proved more reliable than wifi. Some staff eventually educated themselves re technical issues and IT department provided some education, although this was limited. Over time clients and staff became a little more confident re using Telehealth.

1-59	provided ...gifts cards to compensate extra money spent on data; clear group guidelines and expectations set for sessions and revisited as and when needed (addressed as a TIB in individual as well); extra facilitator as a "skills coach" for the first couple of modules that could take someone into a break out room when they were distressed - to allow facilitators to keep teaching skills. Delayed start of the group to allow the team to develop some semblance of structure and confidence facilitating DBT virtually.
1-66	Sometimes we liaised with case managers to help a client upgrade their internet connection. Most importantly, we set up guidelines for "Doing Virtual DBT" . These were rules about the etiquette of teleconferencing and how to prepare for the session. We both mailed and emailed copies of the week's handouts prior to the session. Therapists and clients became more confident and relaxed. Even though spontaneous interactions were curtailed, the more structured process was helpful for teaching the material
3-68	Used zoom chat room function with facilitators dropping into rooms to oversee. Not as efficient or engaging as face to face seemingly but definitely useful. Managing group connectedness was challenging - we provided space to discuss this openly and grieve this lost opportunity. The privacy issue was tricky - we had clear rules around joining from public places which weren't followed at times which led to some disconsternation. A small number identified that they did not get as much out of teleconferenced group as F2F.
1-72	-our [service] loaned dongles for wifi and tablets to clients -we prepared clients the week before about things like TIP and told them what they needed to bring --we have to be very organized and send out the pack of homework several weeks before group to allow for slow post service during covid. We also email it to them
3-104	Access to technology /wifi. Having a private and quiet space (lots of over crowding in peoples homes). Limited Phone credit.
